# Supplementary material for: Evaluation of Apparent Diffusion Coefficient Repeatability and Reproducibility for Preclinical MRIs Using Standardized Procedures and a Diffusion-Weighted Imaging Phantom
Source: Tomography. 2023 Feb 7;9(1):375–86. doi: 10.3390/tomography9010030 (PMC9964373; doi:10.3390/tomography9010030)
Supplement: Supplementary file 1 [file tomography-09-00030-s001.zip › tomography-2118961-supplementary.pdf]

Supplemental Figures for Tomography Special Issue on "Co-Clinical Trials"

Manuscript Title: Evaluation of ADC Repeatability and Reproducibility of Pre-Clinical MRIs Using Standardized Procedures and DWI Phantom

First Author: Dariya Malyarenko

Communicating Author: Thomas L. Chenevert

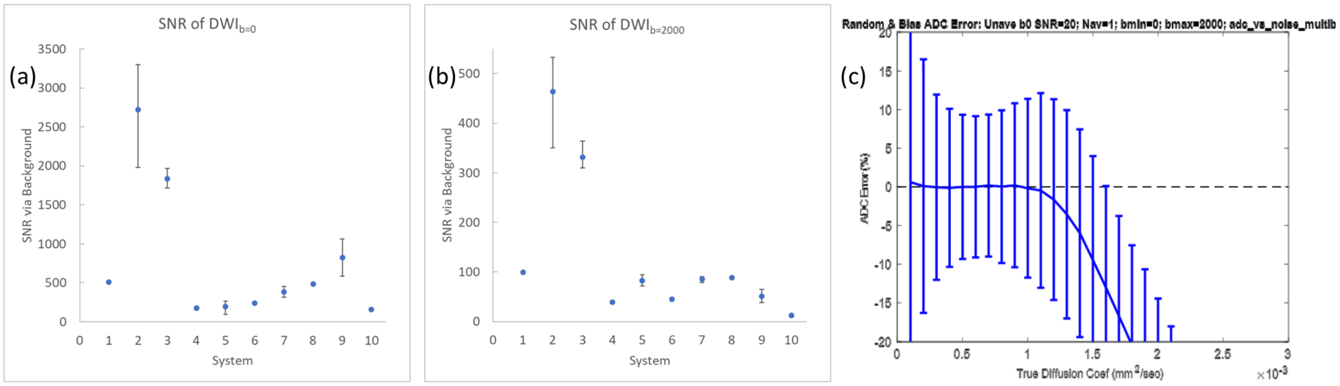

Supplemental Figure S1. Measured SNR at isocenter on ten CIRP MRI systems using (a) low b-value = 0 DWI and (b) high b-value = 2000sec/mm^2. (c) Simulation of random error and bias for the standardized DWI protocol for SNR = 20 (at b=0) corresponding to onset of negative bias for Dtrue = 1.1μm^2/ms. Measured SNR at isocenter exceeded 150 on all CIRP systems, therefore measurements of ADC bias were not significantly affected by low SNR.

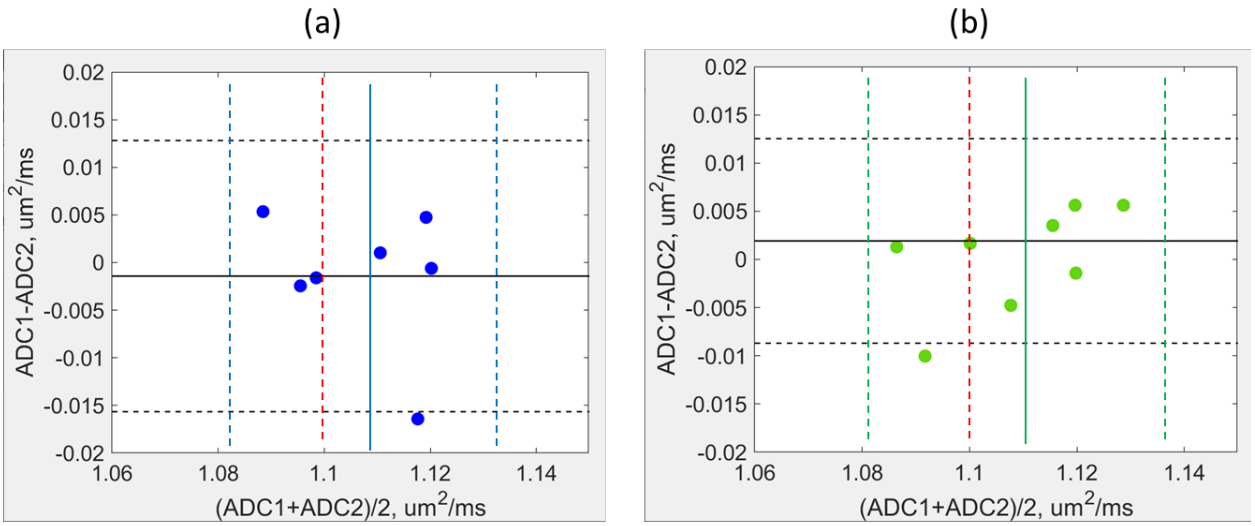

Supplemental Figure S2. Bland-Altman analysis of intra-scan (a) and day-to-day (b) repeatability is shown for isocenter ADC (single slice ROI) of the pre-clinical MRI scanners (excluding outlier systems Sys 9 and Sys 10). For intra-scan repeatability, only the first scan day was used. For inter-scan repeatability, only the first scan of both days was used. Solid horizontal line marks the bias, and dashed lines correspond to 95% limits of agreement (LOA = bias ± 1.96 · SD(ADC1-ADC2)). The averaged observed repeatability was within 0.015 μm^2/ms. With respect to D_{true} = 1.1 μm^2/ms (red vertical dashes), mean across systems ADC (solid vertical lines) exhibits apparent positive bias of 0.007 μm^2/ms in (a) and 0.009 μm^2/ms in (b), and LOA=bias ± (0.025, 0.029) μm^2/ms (dashed vertical lines). The average multi-scanner reproducibility for (a) and (b) is within 0.03 μm^2/ms (3%) with 1% positive bias.
